# Marijuana Use and Health Outcomes in Persons Living With HIV: Protocol for the Marijuana Associated Planning and Long-term Effects (MAPLE) Longitudinal Cohort Study

**DOI:** 10.2196/37153

**Published:** 2022-08-30

**Authors:** Angel B Algarin, Gabriela N Plazarte, Kaitlin R Sovich, Stella D Seeger, Yancheng Li, Ronald A Cohen, Catherine W Striley, Bruce A Goldberger, Yan Wang, Charurut Somboonwit, Gladys E Ibañez, Emma C Spencer, Robert L Cook

**Affiliations:** 1 Division of Infectious Diseases and Global Public Health Department of Medicine University of California, San Diego La Jolla, CA United States; 2 Department of Psychology University of South Florida Tampa, CA United States; 3 Department of Neurology University of Florida Gainesville, FL United States; 4 Department of Epidemiology University of Florida Gainesville, FL United States; 5 Department of Clinical and Health Psychology University of Florida Gainesville, FL United States; 6 Center for Cognitive Aging and Memory University of Florida Gainesville, FL United States; 7 Department of Pathology, Immunology and Laboratory Medicine University of Florida Gainesville, FL United States; 8 Division of Infectious Disease & International Medicine University of South Florida Tampa, FL United States; 9 Department of Epidemiology Florida International University Miami, FL United States; 10 Bureau of Communicable Diseases Florida Department of Health Tallahassee, FL United States

**Keywords:** people living with HIV, marijuana use, cannabis, Florida, longitudinal studies, cognition, protocol

## Abstract

**Background:**

Marijuana use is common in persons with HIV, but there is limited evidence of its relationship with potential health benefits or harms.

**Objective:**

The Marijuana Associated Planning and Long-term Effects (MAPLE) study was designed to evaluate the impact of marijuana use on HIV-related health outcomes, cognitive function, and systemic inflammation.

**Methods:**

The MAPLE study is a longitudinal cohort study of participants living with HIV who were recruited from 3 locations in Florida and were either current marijuana users or never regular marijuana users. At enrollment, participants completed questionnaires that included detailed marijuana use assessments, underwent interviewer-administered neurocognitive assessments, and provided blood and urine samples. Ongoing follow-ups included brief telephone assessments (every 3 months), detailed questionnaires (annually), repeated blood and urine samples (2 years), and linkage to medical records and statewide HIV surveillance data. Supplemental measures related to intracellular RNA, COVID-19, Alzheimer disease, and the gut microbiome were added after study initiation.

**Results:**

The MAPLE study completed enrollment of 333 persons between 2018 and 2021. The majority of participants in the sample were ≥50 years of age (200/333, 60.1%), male (181/333, 54.4%), cisgender men (173/329, 52.6%), non-Hispanic Black (221/333, 66.4%), and self-reported marijuana users (260/333, 78.1%). Participant follow-up was completed in 2022, with annual updates to HIV surveillance data through at least 2027.

**Conclusions:**

The MAPLE study is the largest cohort specifically designed to understand the use of marijuana and its effects on HIV-related outcomes. The study population has significant diversity across age, sex, gender, and race. The data will help clinicians and public health officials to better understand patterns of marijuana use associated with both positive and negative health outcomes, and may inform recommendations for future clinical trials related to medical marijuana and HIV.

**International Registered Report Identifier (IRRID):**

DERR1-10.2196/37153

## Introduction

Marijuana use is common among the more than 1 million people living with HIV in the United States [1]. Between 14% and 56% of people living with HIV report any marijuana use in the past 6 months [2-4], and an increasing proportion of users report daily use and use for medical reasons [3,5-7]. This increase in use corresponds with changing state marijuana laws across the country [8]. In Florida, a state with high incidence and prevalence of HIV infections [1], medical marijuana became legal in 2016 for patients with 10 different health conditions, including HIV [9]. Yet, many people living with HIV continue to use marijuana obtained outside of the legal medical marijuana system, and new evidence is needed to inform health care decisions and policies related to marijuana use in people living with HIV.

The long-term effects of marijuana use on people living with HIV are not clear and could potentially vary across individuals and by variation in cannabinoid type and strength within different strains of marijuana and medical marijuana products. Δ9-tetrahydrocannabinol (THC) is the most psychoactive component of marijuana, whereas some evidence suggests that other components of cannabis, such as cannabidiol (CBD) and various terpenes, or their combinations, show more promise for medical treatments [10,11]. In some studies, marijuana use has been shown to alleviate HIV-related symptoms, such as loss of appetite, fatigue, anxiety, neuropathy, pain, nausea, systemic inflammation, neurocognitive impairment, and gastrointestinal problems, and medication side effects [5,11-19]. However, other studies have found limited, insufficient, or lacking evidence supporting marijuana-related improvements to appetite, anxiety, cognitive functioning [20-22], irritable bowel syndrome, and a variety of neurodegenerative disorders [23]. Additionally, other research highlights inconsistencies in the literature on the association between marijuana use and antiretroviral therapy (ART) use and adherence [24-29]. A Cochrane review found only a few clinical trials related to marijuana, and that “those studies that have been performed have included small numbers of participants and focused on short-term effects. Longer-term data are lacking.” [14].

The HIV National Strategic Plan for 2021-2025 in the United States emphasizes the importance of engagement in care, HIV viral load suppression, and reduction of HIV-associated comorbidities [30], but relatively little is known about the impact of different patterns or motivations related to marijuana use on these outcomes. Therefore, health care providers currently have minimal evidence-based guidance when considering whether to recommend or prescribe marijuana to their patients with HIV/AIDS. Clinicians also lack tools or evidence to help identify which persons are using “too much” marijuana, as evidenced by links to specific behavioral and biological harms or onset of a substance use disorder.

There is also inconsistent evidence regarding the impact of marijuana use on cognitive function [17,20-22]. While acute exposure to marijuana can adversely affect cognitive function, the majority of research has found no significant association of chronic use of marijuana and traditional aspects of cognitive function in older adults living with HIV [31]. Less is known about the relationship of chronic marijuana use with more novel aspects of cognitive function such as planning, prospective memory (remembering what to do in the future), and motivation [31].

Several other aspects of chronic marijuana use in people living with HIV are unknown or understudied, including its association with symptoms of pain and anxiety, problems such as cannabis use disorder, and biological responses such as chronic inflammation [32,33]. There is evidence linking marijuana use with a decreased inflammatory response [11,17,18], but no reports to date have examined this relationship using longitudinal data.

To increase knowledge about the impact of marijuana use on health outcomes among people living with HIV, the Marijuana Associated Planning and Long-term Effects (MAPLE) study was funded by the National Institute on Drug Abuse in 2016. The primary goals of the MAPLE study are to determine the association of different patterns of marijuana use with (1) HIV care engagement, viral suppression, and HIV disease progression; (2) traditional and novel aspects of cognitive function; and (3) biomarkers related to chronic inflammation. This paper describes the study design, research procedures, and modifications to the study after initiation. We also present baseline participant characteristics and discuss some of the challenges encountered during the MAPLE study.

## Methods

### Design/Overview

The MAPLE study is a longitudinal cohort study that aims to evaluate health outcomes in persons with HIV who were exposed or not exposed to marijuana at baseline. The study sought to enroll a diverse sample of persons with HIV, with enrollment occurring from 2018 to 2021. The Southern HIV Alcohol Research Consortium (SHARC) at the University of Florida acts as the central coordinating center. The MAPLE study employed a targeted convenience sampling methodology, with data collected via questionnaires, biological specimens, medical record abstraction, and linkage to existing state HIV surveillance data from the Florida Department of Health (FDOH). The study was designed to have annual assessments with ongoing follow-up via the state surveillance data. Several study procedures were modified after study initiation owing to the coronavirus epidemic and the receipt of 2 funding supplements from the National Institutes of Health (NIH).

### Ethics Approval

The research procedures have been approved by the Institutional Review Boards at the University of Florida (201702564), Florida International University (201702564-IAA), and the FDOH (2018-12UF-UF), and are in accordance with the ethical standards of the responsible committee on human experimentation and with the Helsinki Declaration of 1975.

### Recruitment Settings and Procedures

Recruitment for the MAPLE study began in November 2018 and ended in December 2021. Recruitment was based in the following 3 diverse settings in Florida: Miami, Tampa, and Gainesville. The majority of participants were recruited by research staff based in local county health departments and community-based clinics, but at all sites, participants were also recruited by word of mouth, self-referral, and flyers. Potential participants were screened and those who met preliminary inclusion criteria provided written informed consent, contact information, and confirmation of study eligibility (proof of HIV status and urine cannabinoid test results that matched self-reported use).

### Study Population

Participants were eligible for the MAPLE study if they (1) were 18 years or older; (2) were living with HIV (confirmed by medical records or an HIV medication bottle); (3) were not planning to move out of Florida in the next 12 months; (4) could communicate in English; and (5) had either confirmed marijuana use (cannabinoids urine screen positive and self-reported marijuana use ≥4 times in the past month) or no/very limited marijuana use (cannabinoids urine screen negative, no self-report marijuana use in the past 5 years, and no regular or lifetime use). The planned enrollment (prior to the COVID-19 pandemic) sought to enroll 300 marijuana users and 100 nonusers.

### Baseline Data Collection

#### Study Questionnaire and Assessments

At the baseline visit, participants completed an interviewer-assisted study questionnaire and cognitive assessments via paper, a computer (via Research Electronic Data Capture [REDCap] and Substance Abuse Module), or an iPad, depending on the specific study assessment. The study procedures took approximately 3.5 to 4 hours to complete, and participants received US $75 compensation.

Table 1 lists the study measures, sources, and timepoints for the research questionnaires, which included items to assess demographic characteristics, HIV-related health (eg, history of diagnosis, medication adherence, and retention in care), alcohol and drug use, pain, sleep, sexual behavior, mental health symptoms, interpersonal factors (eg, social support, HIV disclosure, and HIV-related stigma), health care utilization, insurance, and previous incarceration. Additional marijuana-related questions assessed the modes of consumption, reasons for use, and perceived effectiveness. Links to the baseline study questionnaires are included on the SHARC website [34].

**Table 1:** Summary of key measures in the Marijuana Associated Planning and Long-term Effects cohort study.

Key variables		Timepoint			Source
		0 y	1 y	2 y	
Sociodemographics		Yes	Yes	Yes	N/A^a^
**General well-being**					
	Health-related quality of life	Yes	Yes	Yes	Short Form-8 Health Survey [35,36]
	Life goals	Yes	Yes	Yes	Abbreviated Personal Projects Analysis Inventory [37,38]
	Apathy	Yes	Yes	Yes	Apathy Evaluation Scale [39]
	Leisure activities	Yes	Yes	Yes	Adult Leisure Activities [40]
	Hospitalizations (past 12 months)	Yes	Yes	Yes	N/A
**HIV care and treatment**					
	Diagnosis/linkage to care	Yes	No	No	N/A
	ART^b^ use/adherence	Yes	Yes	Yes	Abbreviated ART adherence measure [41]
	Access to care	Yes	Yes	Yes	N/A
	CD4 count/viral load	Yes	Yes	Yes	Enhanced HIV/AIDS reporting system
**Social support**					
	Perceived social support	Yes	Yes	Yes	Medical Outcomes Study – Social Support Survey [42]
	HIV disclosure	Yes	No	No	N/A
	Stigma	Yes	No	No	Modified Berger Stigma Scale [43,44]
**Mental health**					
	Depression	Yes	Yes	Yes	Patient Health Questionnaire-8 [45]
	Anxiety	Yes	Yes	Yes	Generalized Anxiety Disorder-7 [46]
	PTSD^c^	Yes	Yes	Yes	Primary Care PTSD Screen [47]
	Distress	Yes	Yes	Yes	NIH^d^ Distress Thermometer [48]
	Life stress	No	No	Yes	Holmes-Rahe Life Stress Inventory [49]
**Neurocognitive functioning**					
	Premorbid intellectual function	Yes	No	No	Wechsler Test of Adult Reading [50]
	Episodic verbal learning and memory	Yes	Yes^e^	Yes	NIH Toolbox Picture Sequence Memory Test [51-53] California Verbal Learning Test-II [54]
	Prospective memory performance	Yes	Yes^e^	Yes	Memory for Intentions Screening Test [55]
	Planning	Yes	Yes^e^	Yes	NIH Examiner Unstructured Task [56]
	Cognitive, emotional, sensory, and motor functions	Yes	Yes^e^	Yes	NIH Toolbox Dimensional Change Card Sort Test [52,57,58] NIH Toolbox Pattern Comparison Processing Speed Test [52,59] NIH Toolbox Oral Symbol Digit Test [52,60]
	Inhibitory control and attention	Yes	Yes^e^	Yes	NIH Toolbox Flanker Inhibitory Control and Attention Test [52,61,62]
	Working memory	Yes	Yes^e^	Yes	NIH Toolbox List Sorting Working Memory Test [52,63]
**Behavioral risk factors**					
	Substance use	Yes	Yes	Yes	Alcohol Use Disorder Identification Test [64]
	Sexual behavior	Yes	Yes	Yes	Adapted Veterans Aging Cohort Study sexual risk measure [65]
**Other health conditions**					
	Pain	Yes	Yes	Yes	Brief Pain Inventory [66] Short Form McGill Pain Questionnaire-2 [67]
	Sleep	Yes	Yes	Yes	Abbreviated Pittsburgh Sleep Quality Assessment [68]
**Other**					
	Incarceration history	Yes	Yes	Yes	N/A
	Use of technology	Yes	Yes	Yes	N/A
**Marijuana**					
	Information sources	Yes	No	No	N/A
	Attitudes and beliefs	Yes	Yes	Yes	N/A
	Use patterns	Yes	Yes	Yes	Timeline Followback [69,70]
	Cannabis use disorder	Yes	No	No	CIDI-Substance Abuse Module [71,72]
	Past 12-month use disorder	Yes	Yes	Yes	Mini International Neuropscyhiatric Interview [73], Cannabis Intervention Screener [74]
	Reasons for use	Yes	Yes	Yes	N/A
	Feelings and body effects	Yes	Yes	Yes	N/A
	Sources of marijuana	Yes	Yes	Yes	N/A
	Marijuana and driving	No	No	Yes	N/A

^a^N/A: not applicable.

^b^ART: antiretroviral therapy.

^c^PTSD: posttraumatic stress disorder.

^d^NIH: National Institutes of Health.

^e^Data from 57 participants were collected at this point owing to COVID-19 modification.

#### Marijuana Use Items

We developed an interviewer-administered marijuana questionnaire using several items developed in our previous research [6], including items to assess specific reasons for use (eg, to improve sleep and increase appetite); perceived effectiveness; use of prescription drugs, such as dronabinol, CBD, or synthetic marijuana; and interest/participation in a formal medical marijuana program (Table 1).

Data on marijuana quantity, frequency, and use patterns were obtained by the Timeline Followback (TLFB), a retrospective calendar-based measure initially developed for alcohol use, but previously found reliable for use with other substances (eg, cocaine, marijuana, and tobacco) [69,70]. Trained MAPLE research assistants used the TLFB to assess patterns related to the timing of doses, mode of consumption (eg, vaporized, smoked, ingested, etc), quantity (in grams of marijuana flower/weed or mg of THC), and frequency over the past 30 days. A calendar and other prompts, such as pictures of different marijuana products (to help estimate quantity), were used to assist participants. Information on the TLFB will be used to create summary variables such as frequency of use (days/month), quantity/use days, and number of heavy use days (eg, >1 gram flower/day).

Several measures were used to assess cannabis use disorder and related issues. The Substance Abuse Module [71,72], recently updated for the Diagnostic and Statistical Manual of Mental Disorders-5, includes questions about onset and recency of specific symptoms, as well as the specific withdrawal symptoms and physical, social, and psychological consequences for marijuana used by participants. It also includes the quantity and frequency of both the heaviest use and use in the past 12 months, age at first and last use, age at first and most recent symptoms, age at which substance use disorder criteria were first and most recently met, and age at remission. Most participants also completed The Standard Mini-International Neuropsychiatric Interview [73], version 7.0.2, which was adapted for use in this study and used to assess the criteria for cannabis use disorder in the past 12 months. Participants also completed the Cannabis Intervention Screener, a brief screening instrument developed for use in medical and social service settings to identify individuals using cannabis at levels that may impact their health or social functioning [74].

Participants completed a cognitive assessment battery that took approximately 75 minutes, which measured premorbid intelligence; episodic, working, and prospective memory; planning; language; and processing speed. The majority of the cognitive assessments were administered using the NIH Toolbox on a digital tablet [51,52], while other more traditional tests (eg, Wechsler Test of Adult Reading, California Verbal Learning Test, Memory for Intentions Screening Test [MIST], and NIH Examiner) were administered using paper and pencil [50,54-56]. The cognitive assessment also included some more novel assessments, including a “Life Goals Inventory” and an apathy scale (to assess motivation) [39], the NIH Examiner (to assess planning) [56], and the MIST (to assess prospective memory) [55]. More details on specific items and assessments can be found in Table 1.

#### Biospecimens

At baseline, participants provided blood and urine samples. Planned blood tests included HIV viral load, tests of liver function, and tests of biomarkers related to inflammation, including cytokines/chemokines, markers of microbial translocation, and intracellular RNA (Table 2). Urine testing included an 8-drug immunoassay panel at baseline and liquid chromatography-mass spectrometry analysis of cannabinoid analytes in urine as outlined in Table 2.

**Table 2:** Additional data collection.

Source	Timepoint				Data
	0 y	1 y	2 y		
Medical record abstraction	Yes	Yes	Yes	Height, weight, current conditions and medications, and laboratory studies (complete blood count, metabolic panel, and hepatitis C virus [HCV] antibody and viral load)	
Blood	Yes	Yes^a^	Yes	HIV viral load (baseline and follow-up) and HCV antibody and viral load; markers of systemic inflammation (sCD14, sCD163, sCD27, C-reactive protein, interleukin 6, and tumor necrosis factor alpha); markers of microbial translocation (lipopolysaccharide, lipopolysaccharide binding protein, and intestinal fatty-acid binding protein); and intracellular RNA expression	
Urine	Yes	Yes^a^	Yes	Δ9-tetrahydrocannabinol, cannabidiol, cannabinol, cannabigerol, 11-hydroxy-Δ9-tetrahydrocannabinol, 11-nor-9-carboxy-Δ9-tetrahydrocannabinol, creatinine, specific gravity, and dipstick drug screen	

^a^Data from 57 participants were collected at this point owing to COVID-19 modification.

#### Medical Records

At the time of enrollment, participants either brought a copy of their medical records from their care provider (and received an additional US $20 compensation) or completed a Health Insurance Portability and Accountability Act authorization form authorizing access to their medical records. Medical records were abstracted from paper copies mailed from clinics, directly from clinic records, or through direct download of electronic medical record data, using privacy procedures approved by all participating institutional review boards. Using the time period as close as possible to the time of baseline survey completion, research assistants abstracted participants’ current medical problems (with International Classification of Diseases [ICD] 9 or ICD-10 codes), current medications, and laboratory results, including HIV viral load, CD4+ count, hepatitis B and C antibodies and viral loads, complete blood count, and metabolic panel, including liver and kidney function.

#### Linkage to HIV Surveillance Data

The research team established a data use agreement with the FDOH Division of HIV Surveillance, which maintains the Enhanced HIV/AIDS Reporting System (eHARS) database in collaboration with the Centers for Disease Control and Prevention. The eHARS database includes CD4+ count, viral load, and other HIV-related information from all persons with HIV in Florida, with new data uploaded over time [1]. Using a procedure we developed for previous studies [75], the study team worked with the FDOH to confidentially link the eHARS with study participant data every 6 months to allow for ongoing follow-up surveillance. Follow-up with HIV surveillance data will continue for at least 5 years after enrollment.

### Follow-up Assessments

In brief, follow-up phone surveys were attempted for each participant at 3-, 6-, and 9-month time points between each annual visit until recruitment stopped in 2021. These calls sought to improve retention and to capture changes in participants’ marijuana use and health status. The study originally planned to have annual in-person assessments, but due to COVID, the 1-year follow-ups were mostly completed by telephone or videoconference. These assessments included nearly all follow-up questionnaire items and marijuana use assessments including the TLFB, but did not include several of the formal neurocognitive assessments or the collection of blood or urine samples. For the 2-year follow-ups, the majority of participants completed in-person questionnaires and cognitive assessments, and also provided follow-up blood and urine samples. Participants received US $10 incentive payments for each quarterly telephone assessment and US $75 for each annual follow-up study visit.

### Modifications After Study Initiation

#### COVID-19

As a result of the COVID-19 pandemic, study activities were halted between March and June 2020 while the research team modified procedures to allow more remote data collection. Specifically, from June to November 2020, we adapted the 1-year follow-up survey to be collected by telephone and removed the neurocognitive assessments and biospecimen collection from the 1-year follow-up. Some participants were unaffected by this change as their data were collected prior to this time. The study resumed limited recruitment of new participants in November 2020, incorporating a hybrid virtual/in-person experience where the majority of the study questionnaires were completed by phone or video call before the participant’s in-person appointment for laboratory testing, neurocognitive assessments, and the remaining questionnaire items. The study also received additional funding from an NIH-supported COVID supplement to incorporate items to assess how the COVID pandemic influenced marijuana use and overall health in this population and to test for COVID antibodies. Many of these assessments were integrated into the 3-month brief phone assessments. Qualitative interviews of a subset of participants related to their experience with COVID and changes in marijuana use were also completed.

#### Alzheimer Supplement

The study also received additional NIH funding to incorporate some additional items to better understand the relationship of marijuana use with cognitive decline in aging. These study activities were initiated for persons aged 60 years or older starting in October 2019. The addition of this supplement consisted of additional neurocognitive measures that could more formally distinguish mild cognitive impairment and incorporate additional blood testing, and stool sampling was performed for microbiome assessments.

### Quality Assurance and Data Management

#### Quality Assurance

Before study initiation, the research team underwent extensive training to ensure that all assessments were provided following the standard protocol. The central project coordinating team monitored for quality assurance by reviewing recordings of a sample of interviews from each research assistant to ensure standardized study procedures, and by conducting site visits 1 to 2 times a year. At weekly team meetings, the research staff discussed and addressed any protocol deviations and monitored for adverse events.

#### Data Management

The MAPLE database is maintained at the University of Florida on secure servers. REDCap is used to collect and maintain participant contact registry data, enrollment/study visit logs, questionnaire data, and scores from neurologic assessments. All data collected using paper forms have been scanned and uploaded into REDCap. Paper-based assessments were scored by study interviewers and double-checked by the site study coordinator to reduce data errors.

#### Planned Data Analyses

The longitudinal nature of the MAPLE study will allow for both cross-sectional and longitudinal analyses. Cross-sectional analyses will assess the factors associated with different patterns of marijuana use exposure, and the relationship of patterns of marijuana use with outcomes of interest at baseline. For longitudinal analyses, we plan to examine how outcomes of interest (eg, HIV-related outcomes, neurocognitive outcomes, and HIV-related inflammatory biomarkers) vary according to changes in marijuana use (increases or decreases). Additional analyses will consider the impact of lifetime history of marijuana use, the presence of cannabis use disorder, and outcomes related to symptoms such as pain or stress.

### Power Considerations

The research team originally proposed to enroll 400 persons (300 who were current marijuana users), but the COVID-19 epidemic hit at the peak of the enrollment period, and there were significant delays in enrollment for at least 6 months. Therefore, the study co-investigators modified the targeted enrollment to 333, which slightly increased the minimal effect size that could be detected with statistical significance. Specifically, with a 3:1 exposed/nonexposed ratio, 333 participants provide 80% power to detect differences in key outcomes that produce odds ratios (ORs) ranging from 2.2 to 3.6 (whereas 400 persons could have detected ORs ranging from 1.9 to 3.1). For outcomes treated as continuous variables, the current sample can detect a shift of 0.4 SDs with 80% power (whereas 400 persons could have detected a shift of 0.35 SDs).

## Results

### Sample Demographics

Table 3 lists the baseline characteristics of the final sample (N=333). The majority of participants in the sample were ≥50 years of age (200/333, 60.1%), male (181/333, 54.4%), cisgender men (173/329, 52.6%), non-Hispanic Black (221/333, 66.4%), and self-reported marijuana users (260/333, 78.1%). By geographic location, the largest proportion of participants were recruited in Tampa (137/333, 41.1%), followed by Miami (111/333, 33.3%) and then Gainesville (85/333, 25.5%).

**Table 3:** Characteristics of the people living with HIV in the Marijuana Associated Planning and Long-term Effects cohort study at baseline (N=333).

Characteristic		Value	
Age (years), mean (SD)		50 (12)	
**Age group (years) (N=333), n (%)**			
	18-29	26 (7.8)	
	30-39	45 (13.5)	
	40-49	62 (18.6)	
	50-54	67 (20.1)	
	55-59	57 (17.1)	
	60 or older	76 (22.8)	
**Sex at birth (N=333), n (%)**			
	Male	181 (54.4)	
	Female	152 (45.6)	
	Intersex/ambiguous	0 (0.0)	
**Gender identity (N=329), n (%)^a^**			
	Cisgender man	173 (52.6)	
	Cisgender woman	150 (45.6)	
	Transgender woman	6 (1.8)	
	Transgender man	0 (0.0)	
	Nonbinary/gender nonconforming	0 (0.0)	
**Race/ethnicity (N=333), n (%)**			
	Hispanic	44 (13.2)	
	Not Hispanic, White	53 (15.9)	
	Not Hispanic, Black	221 (66.4)	
	Not Hispanic, other	15 (4.5)	
**Education (N=333), n (%)**			
	Less than high school	98 (29.4)	
	High school or equivalent	113 (33.9)	
	Greater than high school	122 (36.6)	
**Location of recruitment (N=333), n (%)**			
	Miami	111 (33.3)	
	Tampa	137 (41.2)	
	Gainesville	85 (25.5)	
**Recruitment year (N=333), n (%)**			
	2018	10 (3.0)	
	2019	252 (75.7)	
	2020	41 (12.3)	
	2021	30 (9.0)	

^a^Four responses were missing as the participants declined to respond.

### Marijuana Use Patterns

The final sample at baseline included 260 of 333 (78%) persons who were current marijuana users and 73 of 333 (22.3%) nonusers confirmed with urine samples (Table 4). Among the current marijuana users, the majority (188/256, 73.4%) first used marijuana before the age of 18 years, and most of the remaining (62/256, 24.2%) first used it before the age of 25 years. Moreover, the majority (208/235, 88.5%) of participants in the study used marijuana flower only. Most obtained marijuana through an illicit (street) source (194/235, 92.1%) and in small quantities (86/194, 44.3%), and only 20 of 255 (7.8%) reported obtaining marijuana from medical dispensaries in Florida. Among those who only used flower, just over half (123/208, 58.1%) used it every day, and about a third of them (69/204, 33.8%) consumed 2 or more grams of flower per use day (Table 4).

**Table 4:** Marijuana use patterns among people living with HIV in the Marijuana Associated Planning and Long-term Effects cohort study at baseline (N=333).

Variable		Value
**Self-reported marijuana use (N=333), n (%)**		
	Yes	260 (78.1)
	No	73 (21.9)
**Tetrahydrocannabinol urine screen results (N=327), n (%)**		
	Positive	254 (77.7)
	Negative	73 (22.3)
**Had a formal medical marijuana card at enrollment (N=255), n (%)**		
	Yes	20 (7.8)
	No	235 (92.2)
**Age (years) at first use of marijuana (N=256), n (%)**		
	<18 years	188 (73.4)
	18-25 years	62 (24.2)
	≥26 years	6 (2.3)
**Type of marijuana used (N=235), n (%)**		
	Flower only	208 (88.5)
	Any nonflower (vape, etc)	27 (11.5)
**Frequency of marijuana use (among flower users only) (N=208), n (%)**		
	Daily	123 (58.1)
	Less than daily	85 (40.9)
**Quantity of marijuana flower used per use/day (among flower users only) (N=204), n (%)**		
	<1 gram	89 (43.6)
	1-2 grams	46 (22.6)
	2-3 grams	33 (16.2)
	3 or more grams	36 (17.6)

### Ancillary Data and Follow-up

Of the 333 baseline participants, 332 (99.7%) had blood tests and 332 (99.7%) had at least one complete neurocognitive assessment. As of the end of 2021, survey data of 320 of 327 (97.9%) participants had been linked to data from the Florida HIV surveillance system, and thus, they have ongoing longitudinal data related to their engagement in care, HIV viral load, and changes in CD4 counts over time. To date, the research team has obtained baseline medical record abstractions from 215 of 333 (64.6%) participants, and additional collection of medical record information is ongoing. Of the 333 participants who completed baseline assessments, 212 (63.7%) completed the 1-year follow-up. Of these, 53 (25.0%) had blood tests and 54 (25.5%) had at least one cognitive assessment. Moreover, of the 333 participants, 178 (53.5%) completed the 2-year follow-up, of whom 149 (83.7%) had blood tests and 168 (94.4%) had at least one cognitive assessment. Overall, 224 (67.3%) participants completed at least one follow-up visit (either the 1-year or 2-year follow-up).

## Discussion

To our knowledge, the MAPLE study is the largest prospective cohort study designed to improve the understanding of the health impacts of marijuana use in people living with HIV. The study population is somewhat unique for a research study related to marijuana and health, because of the broad diversity of adults across age, race/ethnicity, sex, and gender. Overall, the sample of 333 people living with HIV is reasonably representative of people living with HIV in the Southern United States (people living with HIV in the South: 352,323/474,786, 74.2% male; 245,579/475,547, 51.6% Black) [1]. The MAPLE study participants were enrolled between 2018 and 2021, and completed all in-person assessments on or before June 2022. Follow-up for nearly all participants via the statewide HIV surveillance system is ongoing, and this will provide ongoing outcome data related to the HIV care continuum until at least 2027.

One rationale to establish a cohort study is that cross-sectional comparisons of marijuana users and nonusers have several limitations, including issues related to temporality and confounding (pre-existing differences in marijuana users and nonusers). Other cohort studies, not focused on HIV, have examined the relationship of marijuana to health outcomes [76,77]. Moreover, while some cohort studies of persons with HIV have examined the relationship of marijuana use with health outcomes, they have very limited measures of marijuana use exposure [5,31]. The MAPLE study cohort is unique in that the study will obtain detailed measures of marijuana use over time among a diverse sample of adults living with HIV. The study is also unique in its focus on longitudinal outcomes related to the HIV care continuum, biomarkers of systemic inflammation, and novel aspects of cognition, such as planning, motivation, and prospective memory.

One of the major challenges in the study was the measurement of the quantity of marijuana used by participants. Nearly all participants in the MAPLE study reported using marijuana flower that they had obtained from nondispensary sources, and thus, the specific levels of marijuana components, such as THC and CBD, were not known. The research team met regularly to standardize assessments of the quantity and frequency of marijuana flower use. However, specific levels of corresponding cannabinoids, such as THC and CBD, were hard to estimate, and the research team could not legally obtain flower samples from participants for analysis. There are plans to validate the self-reported amounts with urine biomarkers for THC and CBD metabolites.

Participant recruitment and retention were also challenging. Persons were eligible if they were either a current marijuana user or someone who never or only rarely used marijuana in the past. However, many persons who were not current users but had used marijuana regularly in the past were excluded. Recruitment from 3 different settings helped to improve overall generalizability of the sample; yet with multiple settings, staff turnover also occasionally impacted enrollment because of the complexity of the study protocol. The study recruitment was just reaching its peak when the COVID-19 epidemic occurred and halted recruitment for a period during which study procedures were modified and institutional review board protocols were revised. Study follow-up visits during the pandemic also posed challenges. Due to clinic closures and minimal in-person services, some of the key outcome measures that required blood samples or neurocognitive assessments were only obtained in a small proportion of participants at 1 year. Nevertheless, the majority of participants completed at least one full follow-up assessment 1-2 years after enrollment, and this information will support analyses related to changes over time. Missing outcome data will affect the magnitude of the effect that can be detected with statistical significance, especially for the inflammatory and cognitive outcomes, but not for the HIV-related outcomes, which are tracked via the statewide HIV surveillance system.

The planned analyses of the MAPLE study will provide new evidence to improve our understanding of the health effects of marijuana use in persons with a chronic disease such as HIV infection, and some publications using the data are starting to emerge [37,78].

Future research studies may provide even stronger evidence by collecting data before and after the initiation of marijuana use (for persons who are current nonusers), or before and after cessation of marijuana use (for current users). Randomized clinical trials of marijuana and some of its components are also possible, but research studies that involve regular (daily) use in a clinical trial continue to be extremely challenging owing to a variety of legal and regulatory issues. For now, the MAPLE study data are available for sharing with a formal Data Use Agreement, and the process to request data is described on the SHARC website [34].

## Abbreviations

CBD: cannabidiol
eHARS: Enhanced HIV/AIDS Reporting System
FDOH: Florida Department of Health
ICD: International Classification of Diseases
MAPLE: Marijuana Associated Planning and Long-term Effects
MIST: Memory for Intentions Screening Test
NIH: National Institutes of Health
OR: odds ratio
REDCap: Research Electronic Data Capture
SHARC: Southern HIV Alcohol Research Consortium
THC: tetrahydrocannabinol
TLFB: Timeline Followback

## Data Availability

Data can be requested from the Marijuana Associated Planning and Long-term Effects (MAPLE) project through a publicly accessible website hosted by the Southern HIV and Alcohol Research Consortium through their concept system at https://sharc-research.org/research/data/sharc-concepts-system/.

## References

[1] HIV Surveillance Reports. Centers for Disease Control and Prevention.

[2] Mimiaga MJ, Reisner SL, Grasso C, Crane HM, Safren SA, Kitahata MM, Schumacher JE, Mathews WC, Mayer KH (2013). Substance use among HIV-infected patients engaged in primary care in the United States: findings from the Centers for AIDS Research Network of Integrated Clinical Systems Cohort. Am J Public Health.

[3] Pacek LR, Towe SL, Hobkirk AL, Nash D, Goodwin RD (2018). Frequency of cannabis use and medical cannabis use among persons living with HIV in the United States: findings from a nationally representative sample. AIDS Educ Prev.

[4] Thompson AB, Gillespie SE, Hood J, Thomas-Seaton L, Hussen SA, Camacho-Gonzalez AF (2018). Regular marijuana use is associated with poor viral suppression in HIV-infected adolescents and young adults. AIDS Behav.

[5] Dʼsouza G, Matson P, Grady C, Nahvi S, Merenstein D, Weber KM, Greenblatt R, Burian P, Wilson TE (2012). Medicinal and recreational marijuana use among HIV-infected women in the Women's Interagency HIV Study (WIHS) cohort, 1994-2010. J Acquir Immune Defic Syndr.

[6] Mannes Z, Burrell II L, Ferguson E, Zhou Z, Lu H, Somboonwit C, Cook R, Ennis N (2018). The association of therapeutic versus recreational marijuana use and antiretroviral adherence among adults living with HIV in Florida. PPA.

[7] Slawson G, Milloy M, Balneaves L, Simo A, Guillemi S, Hogg R, Montaner J, Wood E, Kerr T (2015). High-intensity cannabis use and adherence to antiretroviral therapy among people who use illicit drugs in a Canadian setting. AIDS Behav.

[8] Yu B, Chen X, Chen X, Yan H (2020). Marijuana legalization and historical trends in marijuana use among US residents aged 12-25: results from the 1979-2016 National Survey on drug use and health. BMC Public Health.

[9] Florida Constitution-Article X Section 29. The Florida Legislature.

[10] Whiting PF, Wolff RF, Deshpande S, Di Nisio M, Duffy S, Hernandez AV, Keurentjes JC, Lang S, Misso K, Ryder S, Schmidlkofer S, Westwood M, Kleijnen J (2015). Cannabinoids for medical use: a systematic review and meta-analysis. JAMA.

[11] Wilson NL, Peterson SN, Ellis RJ (2021). Cannabis and the gut-brain axis communication in HIV infection. Cannabis Cannabinoid Res.

[12] de Jong BC, Prentiss D, McFarland W, Machekano R, Israelski DM (2005). Marijuana use and its association with adherence to antiretroviral therapy among HIV-infected persons with moderate to severe nausea. J Acquir Immune Defic Syndr.

[13] Corless IB, Lindgren T, Holzemer W, Robinson L, Moezzi S, Kirksey K, Coleman C, Tsai Y, Sanzero Eller L, Hamilton MJ, Sefcik EF, Canaval GE, Rivero Mendez M, Kemppainen JK, Bunch EH, Nicholas PK, Nokes KM, Dole P, Reynolds N (2009). Marijuana effectiveness as an HIV self-care strategy. Clin Nurs Res.

[14] Lutge E, Gray A, Siegfried N (2013). The medical use of cannabis for reducing morbidity and mortality in patients with HIV/AIDS. Cochrane Database Syst Rev.

[15] Woolridge E, Barton S, Samuel J, Osorio J, Dougherty A, Holdcroft A (2005). Cannabis use in HIV for pain and other medical symptoms. J Pain Symptom Manage.

[16] National Academies of Sciences, Engineering, and Medicine (2017). The Health Effects of Cannabis and Cannabinoids: The Current State of Evidence and Recommendations for Research.

[17] Watson C, Paolillo E, Morgan E, Umlauf A, Sundermann EE, Ellis RJ, Letendre S, Marcotte TD, Heaton RK, Grant I (2020). Cannabis exposure is associated with a lower likelihood of neurocognitive impairment in people living with HIV. J Acquir Immune Defic Syndr.

[18] Manuzak J, Gott T, Kirkwood J, Coronado E, Hensley-McBain T, Miller C, Cheu RK, Collier AC, Funderburg NT, Martin JN, Wu MC, Isoherranen N, Hunt PW, Klatt NR (2018). Heavy cannabis use associated with reduction in activated and inflammatory immune cell frequencies in antiretroviral therapy-treated human immunodeficiency virus-infected individuals. Clin Infect Dis.

[19] Farokhnia M, McDiarmid GR, Newmeyer MN, Munjal V, Abulseoud OA, Huestis MA, Leggio L (2020). Effects of oral, smoked, and vaporized cannabis on endocrine pathways related to appetite and metabolism: a randomized, double-blind, placebo-controlled, human laboratory study. Transl Psychiatry.

[20] Cristiani SA, Pukay-Martin ND, Bornstein RA (2004). Marijuana use and cognitive function in HIV-infected people. J Neuropsychiatry Clin Neurosci.

[21] Schouten J, Su T, Wit F, Kootstra NA, Caan MWA, Geurtsen GJ, Schmand BA, Stolte IG, Prins M, Majoie CB, Portegies P, Reiss P, AGEhIV Study Group (2016). Determinants of reduced cognitive performance in HIV-1-infected middle-aged men on combination antiretroviral therapy. AIDS.

[22] Skalski LM, Towe SL, Sikkema KJ, Meade CS (2016). The impact of marijuana use on memory in HIV-infected patients: a comprehensive review of the HIV and marijuana literatures. Curr Drug Abuse Rev.

[23] Abrams DI (2018). The therapeutic effects of Cannabis and cannabinoids: An update from the National Academies of Sciences, Engineering and Medicine report. Eur J Intern Med.

[24] Gross IM, Hosek S, Richards MH, Fernandez MI (2016). Predictors and profiles of antiretroviral therapy adherence among African American adolescents and young adult males living with HIV. AIDS Patient Care STDS.

[25] Zhang Y, Wilson TE, Adedimeji A, Merenstein D, Milam J, Cohen J, Cohen M, Golub ET (2018). The impact of substance use on adherence to antiretroviral therapy among HIV-infected women in the United States. AIDS Behav.

[26] Bonn-Miller MO, Oser ML, Bucossi MM, Trafton JA (2014). Cannabis use and HIV antiretroviral therapy adherence and HIV-related symptoms. J Behav Med.

[27] Lake S, Kerr T, Capler R, Shoveller J, Montaner J, Milloy M (2017). High-intensity cannabis use and HIV clinical outcomes among HIV-positive people who use illicit drugs in Vancouver, Canada. Int J Drug Policy.

[28] Okafor CN, Cook RL, Chen X, Surkan PJ, Becker JT, Shoptaw S, Martin E, Plankey MW (2017). Trajectories of marijuana use among HIV-seropositive and HIV-seronegative MSM in the Multicenter AIDS Cohort Study (MACS), 1984-2013. AIDS Behav.

[29] Sinha S, McCaul ME, Hutton HE, Monroe AK, Alvanzo A, Lesko C, Lau B, Keruly J, Moore RD, Chander G (2017). Marijuana use and HIV treatment outcomes among PWH receiving care at an urban HIV clinic. J Subst Abuse Treat.

[30] HIV national strategic plan for the United States: A roadmap to end the epidemic 2021-2025. U.S. Department of Health and Human Services.

[31] Okafor CN, Plankey MW, Li M, Chen X, Surkan PJ, Shoptaw S, Martin E, Cohen R, Sacktor N, Cook RL (2019). Association of marijuana use with changes in cognitive processing speed and flexibility for 17 Years in HIV-seropositive and HIV-seronegative men. Subst Use Misuse.

[32] Turcotte C, Blanchet M, Laviolette M, Flamand N (2016). Impact of cannabis, cannabinoids, and endocannabinoids in the lungs. Front Pharmacol.

[33] Maggirwar SB, Khalsa JH (2021). The link between cannabis Use, immune system, and viral infections. Viruses.

[34] Southern HIV and Alcohol Research Consortium (SHARC).

[35] Ware JE, Kosinski M, Dewey JE, Gandek B (2001). How to Score and Interpret Single-Item Health Status Measures: A Manual for Users of the SF-8 Health Survey.

[36] Lefante JJ, Harmon GN, Ashby KM, Barnard D, Webber LS (2005). Use of the SF-8 to assess health-related quality of life for a chronically ill, low-income population participating in the Central Louisiana Medication Access Program (CMAP). Qual Life Res.

[37] Algarin AB, Li Y, Cohen RA, Cook CL, Brumback B, Cook RL, Ibañez GE (2021). HIV-related stigma and life goals among people living with HIV (PLWH) in Florida. Qual Life Res.

[38] Little BR (2016). Personal projects. Environment and Behavior.

[39] Marin RS, Biedrzycki RC, Firinciogullari S (1991). Reliability and validity of the apathy evaluation scale. Psychiatry Research.

[40] Jopp DS, Hertzog C (2010). Assessing adult leisure activities: an extension of a self-report activity questionnaire. Psychol Assess.

[41] Wilson IB, Fowler FJ, Cosenza CA, Michaud J, Bentkover J, Rana A, Kogelman L, Rogers WH (2014). Cognitive and field testing of a new set of medication adherence self-report items for HIV care. AIDS Behav.

[42] Sherbourne CD, Stewart AL (1991). The MOS social support survey. Social Science & Medicine.

[43] Wright K, Naar-King S, Lam P, Templin T, Frey M (2007). Stigma scale revised: reliability and validity of a brief measure of stigma for HIV+ youth. J Adolesc Health.

[44] Williams R, Cook R, Brumback B, Cook C, Ezenwa M, Spencer E, Lucero R (2020). The relationship between individual characteristics and HIV-related stigma in adults living with HIV: medical monitoring project, Florida, 2015-2016. BMC Public Health.

[45] Kroenke K, Strine TW, Spitzer RL, Williams JB, Berry JT, Mokdad AH (2009). The PHQ-8 as a measure of current depression in the general population. J Affect Disord.

[46] Spitzer RL, Kroenke K, Williams JBW, Löwe B (2006). A brief measure for assessing generalized anxiety disorder: the GAD-7. Arch Intern Med.

[47] Kimerling R, Trafton JA, Nguyen B (2006). Validation of a brief screen for Post-Traumatic Stress Disorder with substance use disorder patients. Addict Behav.

[48] Ownby K (2019). Use of the distress thermometer in clinical practice. JADPRO.

[49] Noone P (2017). The Holmes-Rahe Stress Inventory. Occup Med (Lond).

[50] Wechsler D (2001). Wechsler Test of Adult Reading: WTAR.

[51] Bauer PJ, Dikmen SS, Heaton RK, Mungas D, Slotkin J, Beaumont JL (2013). III. NIH Toolbox Cognition Battery (CB): measuring episodic memory. Monogr Soc Res Child Dev.

[52] Kallen M, Slotkin J, Griffith J, Magasi S, Salsman J, Nowinski C, Gershon R (2012). NIH Toolbox Technical Manual.

[53] Dikmen SS, Bauer PJ, Weintraub S, Mungas D, Slotkin J, Beaumont JL, Gershon R, Temkin NR, Heaton RK (2014). Measuring episodic memory across the lifespan: NIH toolbox picture sequence memory test. J Int Neuropsychol Soc.

[54] Donders J (2008). A confirmatory factor analysis of the California Verbal Learning Test--Second Edition (CVLT-II) in the standardization sample. Assessment.

[55] Raskin S, Buckheit C, Sherrod C (2010). Memory for Intentions Screening Test: Manual.

[56] Kramer JH, Mungas D, Possin KL, Rankin KP, Boxer AL, Rosen HJ, Bostrom A, Sinha L, Berhel A, Widmeyer M (2013). NIH EXAMINER: Conceptualization and development of an executive function battery. J Int Neuropsychol Soc.

[57] Zelazo PD (2006). The Dimensional Change Card Sort (DCCS): a method of assessing executive function in children. Nat Protoc.

[58] Frye D, Zelazo PD, Palfai T (1995). Theory of mind and rule-based reasoning. Cognitive Development.

[59] Carlozzi NE, Beaumont JL, Tulsky DS, Gershon RC (2015). The NIH Toolbox Pattern Comparison Processing Speed Test: normative data. Arch Clin Neuropsychol.

[60] Smith A (1973). Symbol Digit Modalities Test.

[61] Rueda M, Fan J, McCandliss BD, Halparin JD, Gruber DB, Lercari LP, Posner MI (2004). Development of attentional networks in childhood. Neuropsychologia.

[62] Zelazo PD, Anderson JE, Richler J, Wallner-Allen K, Beaumont JL, Weintraub S (2013). II. NIH Toolbox Cognition Battery (CB): measuring executive function and attention. Monogr Soc Res Child Dev.

[63] Tulsky DS, Carlozzi N, Chiaravalloti ND, Beaumont JL, Kisala PA, Mungas D, Conway K, Gershon R (2014). NIH Toolbox Cognition Battery (NIHTB-CB): List sorting test to measure working memory. J Int Neuropsychol Soc.

[64] Bush K, Kivlahan DR, McDonell MB, Fihn SD, Bradley KA (1998). The AUDIT alcohol consumption questions (AUDIT-C): an effective brief screening test for problem drinking. Ambulatory Care Quality Improvement Project (ACQUIP). Alcohol Use Disorders Identification Test. Arch Intern Med.

[65] Justice A, Dombrowski E, Conigliaro J, Fultz SL, Gibson D, Madenwald T, Goulet J, Simberkoff M, Butt AA, Rimland D, Rodriguez-Barradas MC, Gibert CL, Oursler KAK, Brown S, Leaf DA, Goetz MB, Bryant K (2006). Veterans Aging Cohort Study (VACS): Overview and description. Med Care.

[66] Cleeland CS, Ryan KM (1994). Pain assessment: global use of the Brief Pain Inventory. Ann Acad Med Singap.

[67] Dworkin R, Turk D, Revicki D, Harding G, Coyne KS, Peirce-Sandner S, Bhagwat D, Everton D, Burke LB, Cowan P, Farrar JT, Hertz S, Max MB, Rappaport BA, Melzack R (2009). Development and initial validation of an expanded and revised version of the Short-form McGill Pain Questionnaire (SF-MPQ-2). Pain.

[68] Buysse DJ, Reynolds CF, Monk TH, Hoch CC, Yeager AL, Kupfer DJ (1991). Quantification of subjective sleep quality in healthy elderly men and women using the Pittsburgh Sleep Quality Index (PSQI). Sleep.

[69] Hjorthøj CR, Hjorthøj AR, Nordentoft M (2012). Validity of Timeline Follow-Back for self-reported use of cannabis and other illicit substances--systematic review and meta-analysis. Addict Behav.

[70] Robinson SM, Sobell LC, Sobell MB, Leo GI (2014). Reliability of the Timeline Followback for cocaine, cannabis, and cigarette use. Psychol Addict Behav.

[71] Cottler LB, Compton W (1993). Advantages of the CIDI family of instruments in epidemiological research of substance use disorders. International Journal of Methods in Psychiatric Research.

[72] Cottler LB, Robins LN, Helzer JE (1989). The reliability of the CIDI-SAM: a comprehensive substance abuse interview. Br J Addict.

[73] Lecrubier Y, Sheehan D, Weiller E, Amorim P, Bonora I, Sheehan KH, Janavs J, Dunbar G (2020). The Mini International Neuropsychiatric Interview (MINI). A short diagnostic structured interview: reliability and validity according to the CIDI. Eur. Psychiatr.

[74] Cannabis Intervention Screener. Center for Behavioral Health Integration.

[75] Ibañez GE, Zhou Z, Cook CL, Slade TA, Somboonwit C, Morano J, Harman J, Bryant K, Whitehead NE, Brumback B, Algarin AB, Spencer EC, Cook RL (2021). The Florida Cohort study: methodology, initial findings and lessons learned from a multisite cohort of people living with HIV in Florida. AIDS Care.

[76] Callaghan RC, Allebeck P, Sidorchuk A (2013). Marijuana use and risk of lung cancer: a 40-year cohort study. Cancer Causes Control.

[77] Klebanoff MA, Wilkins DG, Keim SA (2021). Marijuana use during pregnancy and preterm birth: a prospective cohort study. Am J Perinatol.

[78] Wang Y, Ibañez GE, Vaddiparti K, Stetten NE, Sajdeya R, Porges EC, Cohen RA, Cook RL (2021). Change in marijuana use and its associated factors among persons living with HIV (PLWH) during the COVID-19 pandemic: Findings from a prospective cohort. Drug Alcohol Depend.

